# The SAR Payload Design and Performance for the GF-3 Mission

**DOI:** 10.3390/s17102419

**Published:** 2017-10-23

**Authors:** Jili Sun, Weidong Yu, Yunkai Deng

**Affiliations:** Institute of Electronics, Chinese Academy of Sciences, Beijing 100190, China; ywd@mail.ie.ac.cn (W.Y.); ykdeng@mail.ie.ac.cn (Y.D.)

**Keywords:** GF-3, SAR payload, polarization, requirement, working mode, antenna model

## Abstract

This paper describes the C-band multi-polarization Synthetic Aperture Radar (SAR) sensor for the Gaofen-3 (GF-3) mission. Based on the requirement analysis, the design of working modes and SAR payload are given. An accurate antenna model is introduced for the pattern optimization and SAR performance calculation. The paper concludes with an overview of predicted performance which was verified by in-orbit tests.

## 1. Introduction

Gaofen is one program announced in a 15-year plan of Chinese national science and technology programs between 2006 and 2020. Within the Gaofen program, GF-3 is a spaceborne multi-polarization imaging radar mission in C-band.

The GF-3 satellite is circling the Earth in a sun-synchronous dusk-dawn orbit at a 755 km altitude with an inclination of 98.411°, the repeat cycle of orbit is about 29 days. With its multi-polarization C-band Synthetic Aperture Radar (SAR) sensor, GF-3 is able to image the Earth’s surface in all weather conditions and regardless of day or night [1,2].

GF-3 is designed for many areas which include sea and ocean monitoring, disaster reduction, water conservancy, and meteorology. Its primary users are the State Oceanic Administration (SOA), the China Meteorological Administration (CMA), the Ministry of Civil Affairs, and the Ministry of Water Resources. A wide variety of data products with different image characteristics are required by the different users, which introduces great challenges in the SAR system design.

Section 2 contains an overview and analysis of the multi-user requirements for GF-3. Section 3 describes the working mode design, followed by the SAR payload design in Section 4. In Section 5, an accurate antenna model is discussed. The performance of the SAR sensor is presented in Section 6, and conclusions are given in Section 7.

## 2. Requirements

In order to meet the multi-user technical requirements, twelve observing modes were proposed, as listed in Table 1.

The resolution varies from 1 m to 500 m, and the swath varies from 10 km to 650 km. The SAR payload has to support operation in single-polarization (HH or VV), dual-polarization (HH+HV or VH+VV), and quad-polarization (HH+HV+VH+VV) with respect to the observing modes. These characteristics, which vary largely due to multi-user requirements, present great challenges to the SAR system design.

To obtain 1 m azimuth resolution in P mode, sliding spotlight technology is utilized, which means the SAR payload has to provide fast antenna beam steering in the azimuth. The large swath modes have to be implemented as ScanSAR modes which means a fast beam scanning capability in elevation. To obtain 3 m azimuth resolution and continuous measurements in the UF mode, the equivalent azimuth antenna size should be less than 6 m according to the SAR imaging principle. To meet the requirement of high Doppler bandwidth sampling and ambiguity in UF mode, the SAR payload has to transmit in one beam and receive in two beams with different phase centers. In the E mode, the incidence angle is out of the scale of other modes, i.e., larger beam scan angle in the case of fixed embedded antenna. In this case, the sidelobes of the antenna beams have to be low enough to satisfy the ambiguity requirement.

Considering all items listed above, an active phased array antenna is employed, with a 15 m (azimuth) × 1.232 m (elevation). This allows the fast scanning of the antenna beam both in elevation and azimuth, and it can obtain low sidelobes in the case of large scan angle by amplitude and phase optimization. Two Rx channels are necessary for the dual-polarization modes and time-division quad-polarization modes, as well as the single-polarization dual-beam receiving mode, i.e., the UF mode. A microwave switch unit is needed for switching the input signal from the antenna to the two Rx channels.

## 3. Working Modes

### 3.1. Observing Modes

From the perspective of SAR imaging technology, the 12 observing modes can be classified into six groups.

#### 3.1.1. P Mode

P mode employs sliding spotlight [3] technology to raise the azimuth resolution (1 m) at the cost of observing discontinuity. In this mode, only half of the whole antenna aperture in the azimuth, i.e., 7.5 m, is used, which can be any two adjacent panels (A + B or B + C or C + D, which will be described in Section 4.1 and Section 4.2), and in the meanwhile, the other two panels are powered off to reduce the power consumption. With the maximum beam scanning angle of ± 1.9°, a 1 m resolution in the 10 km azimuth imaging length is guaranteed. In elevation, chirp signal with 240 MHz bandwidth achieves 2.29 m~0.98 m ground resolution with respect to 20°~50° incidence angle.

#### 3.1.2. UF Mode

UF mode works in the single-transmit multiple-receive (SIMO) [4] method to reduce the minimum Pulse Repeat Frequency (PRF) needed for Doppler bandwidth, consequently improving the ambiguity. Only half of the whole antenna aperture in the azimuth is used, the same as in P mode, but some differences exist in detail of usage. In UF mode, B + C antenna panels are used in transmitting power, and the azimuth width of the transmitting beam is broadened 1.7 times to fit the receiving beam. B panel and C panel receive backscattering signals separately, which form a two-beam receiving mechanism in order to reduce PRF by means of increasing space sampling. Accordingly, the two Rx channels are supposed to be switched to the signals of two antenna panels in the same polarization.

#### 3.1.3. Quad-Polarization Stripmap Modes

**Figure 1:**
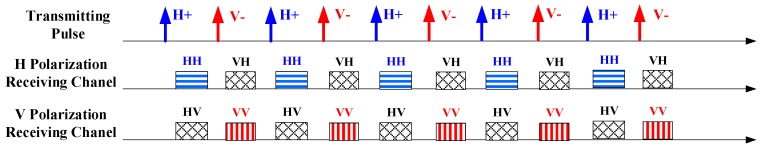
Time-division operation architecture with Inverse modulation slopes.

The quad-polarization stripmap [5] modes, i.e., Q and WQ modes, work in time-division operation architecture [6,7]. The SAR system transmits Horizontal (H) or Vertical (V) polarization pulses in alternate Pulse Repeat Frequency (PRF), whereas it receives backscattering signals in both H and V polarizations. Furthermore, inverse modulation slopes are employed in chirp signal transmitting of H and V polarization. As shown in Figure 1, the H polarization transmitting signal is modulated in the positive slope, and, correspondingly, the V polarization is modulated in the negative slope. The inverse slopes in signal modulation can effectively suppress the cross-polarization ambiguity generated by strong back-scattering ground objects.

All antenna aperture is used in transmitting and receiving. The differences in realization between Q and WQ modes are the partial parameters used in measurement, such as beam pointing and width, signal bandwidth, and looks in image processing with respect to the resolution and swath requirements of the two modes.

#### 3.1.4. WV Mode

WV mode also works in time-division quad-polarization operation architecture, but is different in measurement continuity from the other two quad-polarization modes (Q, WQ). It measures a series of small square areas which are dispersed along one or two incidence angles [8,9]. WV mode is actually a discrete sample mode for a large-scale homogeneous earth surface, generally the sea surface. The SAR payload can work in WV mode for more than 50 min in one acquisition because its discontinuous operation reduces the average power and data column tremendously. WV mode uses the same antenna beams as Q mode. Measurement duration and acquisition time are precisely controlled according to the size of the single observing area (5 km × 5 km normally) and spacing between neighboring areas (50 km normally).

#### 3.1.5. Dual-Polarization Stripmap Modes

The F, WF, S, E modes obtain dual-polarization measurements in the stripmap method. The main differences among these modes are parameters used in measurement, including beam pointing and width, signal bandwidth, and looks in image processing with respect to the resolution and swath requirements. E mode is special because its incidence angle is out of the scale of other modes, which can expand the observing capability to some extent in order to reduce the revisiting interval time.

#### 3.1.6. ScanSAR Modes

The NS, WS, G modes work in the ScanSAR [10] method to cover a larger swath. They use the same antenna beams as the S mode which covers an approximately 130 km swath in every beam. Considering the overlap between adjacent beams, NS, WS, and G modes employ three, five, and seven adjacent beams, respectively, to compose the required swath. It is worth mentioning that G mode can work for more than 30 min in one acquisition, since the power consumption is reduced considerably by cutting down the transmitting duty cycle (about 1.6%), and the data rate is very low by using 2 MHz signal bandwidth.

### 3.2. Inner Calibration Modes

Besides these observing modes, several inner calibration modes are designed to acquire the performance and operating states of the SAR payload. Unlike the external calibration, the inner calibration is supposed to be carried out before and after every observing operation, and even be interjected into the midst of the observing operation, which means monitoring the performance of the SAR payload more frequently.

## 4. SAR Payload Design

### 4.1. Overview

Based on the implementation details of working mode designs, SAR payload is designed. Table 2 provides a brief overview of SAR payload key parameters.

**Figure 2:**
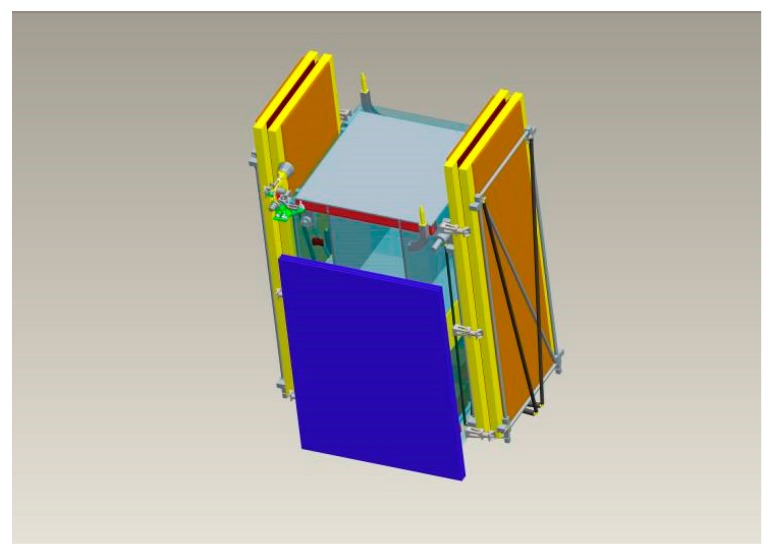
SAR antenna in stowed configuration.

The antenna was divided into four panels which can be powered up separately in order to meet the demand of antenna aperture size coming from the complicated observing modes. The four panels are mounted in a folded configuration at the two opposite sides of the spacecraft before launch, as shown in Figure 2.

After launching and orbit adjusting, a deployable mechanism deploys the panels from a stowed launch configuration into its deployed in-orbit configuration. Before SAR operation, the antenna has to point to −31.5° (right-looking) or +31.5° (left-looking) relying on the roll steering of the satellite platform; this dual-side looking capacity is valuable in reducing the revisit interval time. Zero Doppler steering [11,12,13], which employs yaw and pitch steering to eliminate doppler center frequency shift, was introduced to GF-3.

The SAR payload of the GF-3 satellite are composed of two major subsystems: the antenna subsystem and the electronics subsystem.

### 4.2. SAR Antenna Subsystem

SAR payload employs a deployable planar active phased array antenna [14] which is 15 m (azimuth) × 1.232 m (elevation) in size. The antenna consists of 1536 transmit-receive channels, which are organized in 24 columns (azimuth) and 64 rows (elevation), and distributed over four panels. 

**Figure 3:**
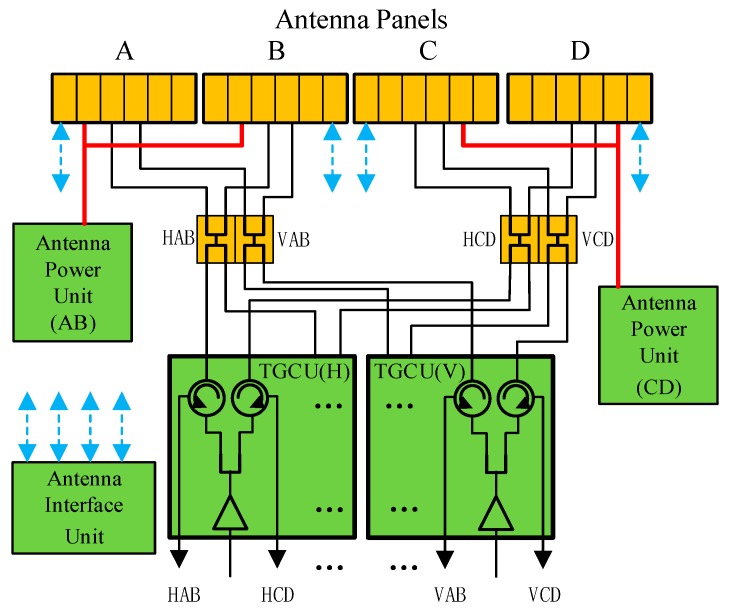
The electric block diagram of the SAR antenna subsystem.

Figure 3 shows the electric block diagram of the SAR antenna subsystem.

The power of the whole antenna is supplied by two Antenna Power Units (APU), which distribute a power bus to four antenna panels and control the aperture configuration by enabling or disabling the direct current (DC) converter in each panel.

The Antenna Interface Unit (AIU) controls all antenna beam forming and steering functions by receiving commands from the electronics subsystem, interpreting the commands, and distributing the beam control commands to four panels in the form of data bus. The phase and gain settings of all elevation beams, including 205 right-looking and 205 left-looking beams, are stored in AIU. The AIU obtains the phase and gain settings of the azimuth steering beam by means of in-orbit calculation.

Transmitting Gain and Circulator Units (TGCU) amplify the transmitting signals received from electronics subsystem to drive the antenna, receive signals from the antenna, and send them to the electronics subsystem. Circulators are involved to separate the transmitting and receiving routes interfacing with the electronics subsystem. Two TGCUs are used to drive and receive from the antenna for H and V polarization respectively.

When transmitting, the signal waveform is amplified by the Radio Frequency (RF) power amplifier and split by a 1:2 divider in TGCU to drive the H or V polarization antenna. The selection of transmitting polarization can be easily applied by enabling the corresponding amplifier located in TGCU. The polarization selection of antenna panels by timing control is also necessary since it can raise the cross-polarization isolation. The H or V polarization signals received from the A and B panels are combined, named HAB or VAB, and then split into two signals, in order to form redundant RF routes to raise the system reliability, as well as signals from the C and D panels.

**Figure 4:**
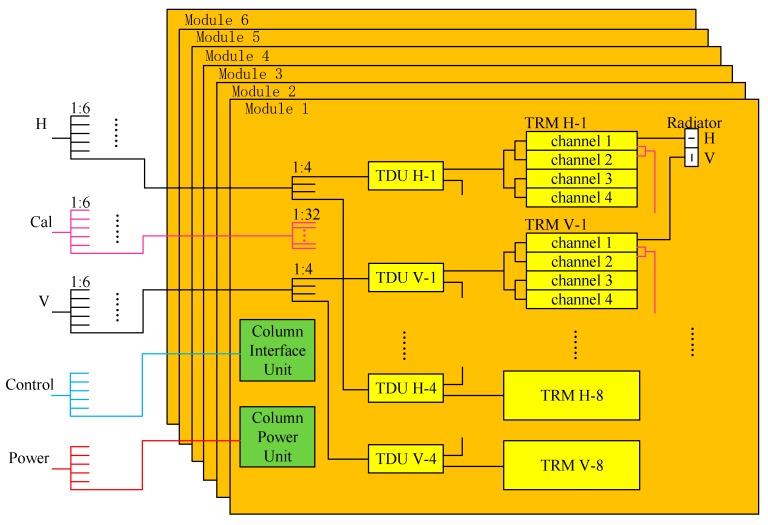
The electric block diagram of one antenna panel.

An electrical block diagram of one antenna panel is given in Figure 4. At the panel level, RF interconnection between the columns and the rest of the radar is provided by three identical 1:6 power dividers. The control bus and power bus are distributed from panel ports to six column modules.

The antenna is a modulated design that defines a column as a standard module, which consists of one Column Interface Unit (CIU), one Column Power Unit (CPU), eight Time Delay Units (TDU), 16 Transmit-Receive Modules (TRM), 32 dual-polarization radiators, three power dividers, and the elevation plane distribution network.

At the column module level, two 1:4 and one 1:32 power dividers are used to interface with the rest of the antenna panel in the RF path. The 1:4 divider distributes H or V signals to the TDU for transmission, and combines the received signals from the TDU ports into H or V output column RF ports. The 1:32 divider is placed between the TRM calibration ports and the column calibration port to distribute or combine H and V inner calibration signals.

Four Transmit-Receive (TR) channels are embedded in one TRM with only one interface with the TDU. Each patch radiator has H and V feeds, which are completely separate on transmitting and signal reception, and connects with two TR channels in H and V polarization, respectively.

The waveguide slot radiator [15] is used to reduce cross polarization and raise radiation efficiency. The selected row spacing of the radiator enables the antenna with a ±20° scanning angle in elevation without exciting grating lobes, which is sufficient and necessary for covering the look angle requirement by twelve observing modes, especially E mode. However, column spacing of the radiator is hard to fit the strict requirements of low grating lobes in the case of a ±1.9° scanning angle in the azimuth needed by the P mode. Fortunately, the grating lobe can be kept from deteriorating in azimuth ambiguity by selecting the proper PRF.

CIU receives commands and timing signals of H and V polarization from AIU. After being interpreted and converted, commands and timing signals are issued to 8 TDUs and 16 TRMs to control beam formation and the transmit-receive switch. CPU converts high-voltage DC power to low-voltage DC power, and distributes it to all active units in the column, including TDUs, TRMs, CIUs.

### 4.3. SAR Electronics Subsystem

The SAR electronics subsystem has the redundant design in most components, it provides the following functions:

- Redundancy control, scheduling, mode definition, timing control;
- Transmitted signal generation;
- Received signal conditioning;
- Quantization, data compression, data formatting.

**Figure 5:**
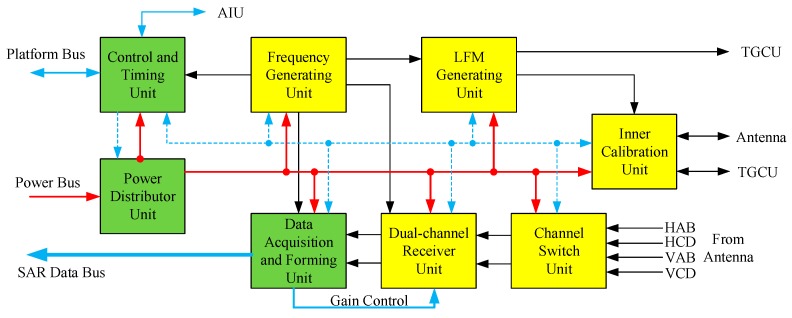
The electric block diagram of the SAR electrics subsystem.

Figure 5 gives an electric diagram of the SAR electronics subsystem.

By means of the communication bus, the Control and Timing Unit (CTU) receives commands from the satellite platform and sends state monitoring data to it. CTU controls the powering up, timing sequence, observing process, and configuration of the SAR payload. In the meantime, the AIU monitors the operating state of the electronics subsystem and the operating state of the antenna subsystem through a specialized data bus.

The Power Distributor Unit (PDU) distributes the platform power bus to all units of the SAR electronics subsystem, as well as AIU and TGCUs of the antenna subsystem, and powers up these units under the control of the CTU and satellite platform. Considering the various demands of the secondary power supply, the DC-DC converters are scattered in the other units instead of being assembled in the PDU.

Based on a high stability oscillator, a Frequency Generating Unit (FGU) generates the synchronous reference frequency signals needed by other units. According to the pulse width, bandwidth, modulation slope, and timing control received from the CTU, the Linear frequency modulated signal Generating Unit (LGU) employs in-orbit real-time calculations to generate the chirp signal waveforms and sends it to the TGCU and Inner Calibration Unit (ICU) after modulation, up-conversion, filtering, and amplifying. On account of the multiple bandwidth demands, eight-bandwidth filtering is supported by the filter bank in LGU.

The four RF signals (HAB, HCD, VAB, and VCD) received from the antenna system are selected or combined in the Channel Switch Unit (CSU) according to the observing modes. In the UF mode, HAB and HCD are selected as the two output signals of the CSU when observing in H single polarization, as well as VAB and VCD in V polarization. In other modes, HAB and HCD are combined into one output of CSU, as well as VAB and VCD are combined to be another one.

Containing two parallel receiver channels, the Dual-channel Receiver Unit (DRU) down-converts the two RF signals into Intermediate Frequency (IF) with selected gain which is controlled by the Data Acquisition and Forming Unit (DAFU). The gain of the DRU can be manually controlled, which means the gain setting responds to the ground command, or can be automatically controlled, which means the settings are adjusted automatically according to the in-orbit calculation results of the DAFU. The same IF filter banks as used in LGU are employed in DRU to cope with different bandwidth signals.

The IF signals are brought into analog-to-digital conversion directly in the DAFU with a sampling frequency of 533.33 MHz [16]. Signal demodulation and filtering are implemented in the digital domain. Down sampling is applied to cut down the SAR raw data rate in case of low signal bandwidth. The two data streams are compressed using 3-bit or 4-bit Block Adaptive Quantization (BAQ) algorithms or a high 4-bit interception algorithm. The selection of compression algorithms as well as the acquisition start time and duration is controlled by the CTU, which interprets the observation commands received from the satellite platform. The compressed data and auxiliary data are formatted according to the observing mode and compression method to form the SAR raw data package. In the inner calibration modes, the auxiliary data and the uncompressed data, which contain the replica of the transmitted waveform, are formatted to form the SAR raw data package.

Inner calibration is carried out in the SAR payload by forming transmit-receive closed RF loops which are achieved by the Inner Calibration Unit (ICU) and the calibration network. The ICU has RF switches, delayed and non-delayed RF paths, and interfaces with the LGU, antenna, and the TGCU. RF loops are formed and switched by controlling the state of the RF switches in the ICU.

## 5. Antenna Model

**Figure 6:**
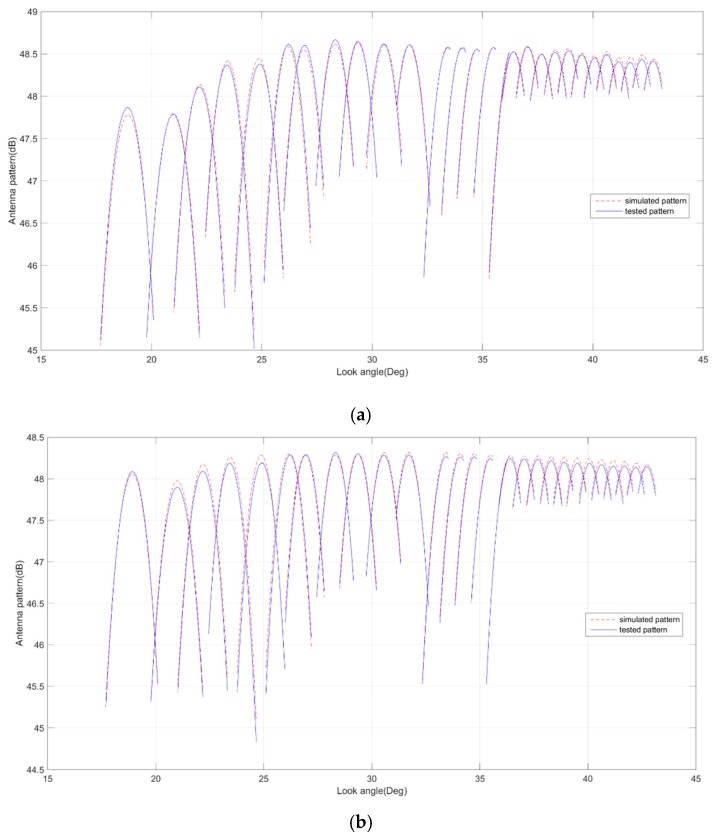
Comparison of simulated and tested patterns of Q mode within the valid main lobe range. (**a**) H-polarization transmitting beams; (**b**) H-polarization receiving beams.

**Figure 7:**
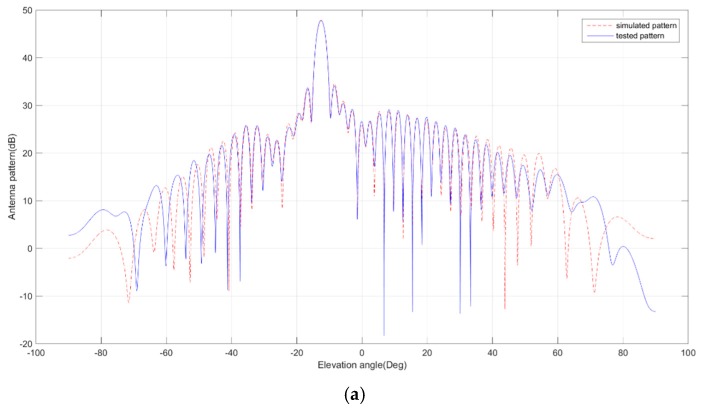

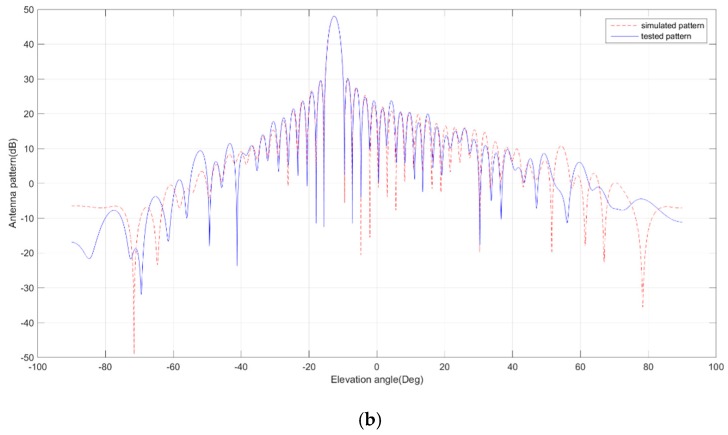
Comparison of simulated and tested patterns of Q1 beam within the full elevation range. (**a**) H-polarization transmitting patterns; (**b**) H-polarization receiving patterns.

Before the antenna patterns were tested, they were simulated using a detailed antenna electric model which was built on the basis of component test data. The model was used in beam optimization to obtain better SAR performance. In the antenna pattern test stage, the model was verified and amended by comparing the tested patterns and the simulated patterns. Figure 6 gives the comparison of the simulated patterns and the tested patterns of Q mode beams in H polarization, which shows maximum 0.2 dB deviation exists within the valid main lobe range corresponding to the imaging area. Moreover, Figure 7 gives the comparison of the simulated patterns and the tested patterns of the Q1 beam in H polarization, which shows good consistency within the full elevation range.

The accurate electric model of the antenna is not only advantageous to the beam optimization, but also to reducing the test time of the antenna patterns as the beams are quite a few due to the 12 observing modes. In the future, it would be useful to carry out some experiment modes in orbit. The performance of experiment modes can be calculated using this antenna model. Based on the good consistency, the antenna pattern simulation and the configuration code generation can be implemented on the ground, since the antenna patterns cannot be tested practically after satellite launching.

## 6. Performance

The predicted performance of the SAR sensor was determined by simulation in the system design stage. Based on the ground test results of the SAR payload, a more accurate performance was calculated before satellite launching, which was verified by in-orbit tests and evaluations, as shown in Table 3.

The azimuth and range ambiguity calculated in the 2D distributed target model is guaranteed by the selected antenna size and beam optimization. The thorough separation of RF routes in H and V polarization raises the polarization isolation performance. Low receiving noise figure, high-efficiency radiator, sufficient antenna gain, and high emission power all contribute to the NESZ. The properties listed in Table 3 are the worst case scenarios in the corresponding incidence scale of the observing modes.

## 7. Conclusions

The GF-3 satellite was launched from the Taiyuan Satellite Launch Centre on 10 August 2016. The first SAR observation was on 15 August 2016. Subsequently, in-orbit performance evaluation and product application tests were gradually carried out. GF-3 has been in operational application officially since January 2017.

**Figure 8:**
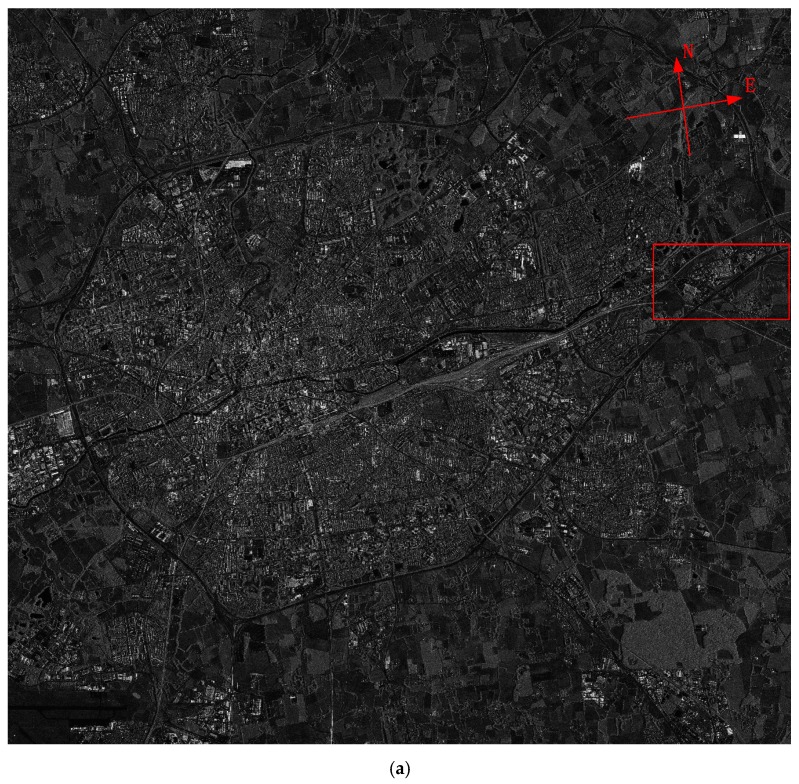

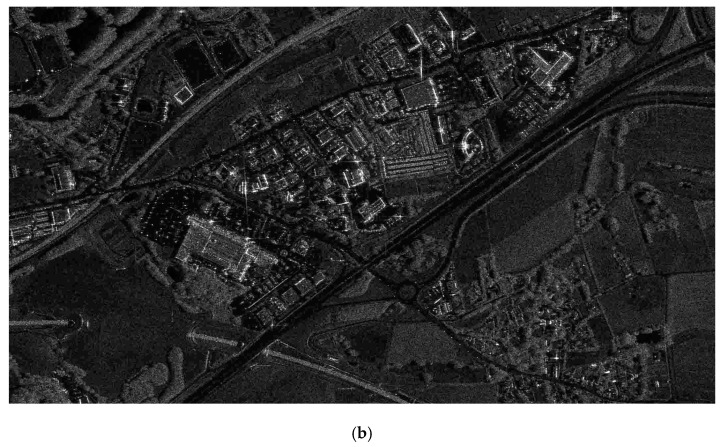
P-mode image of Rennes city, France. (**a**) Whole image; (**b**) Partial enlarged view of red rectangle area in (**a**).

**Figure 9:**
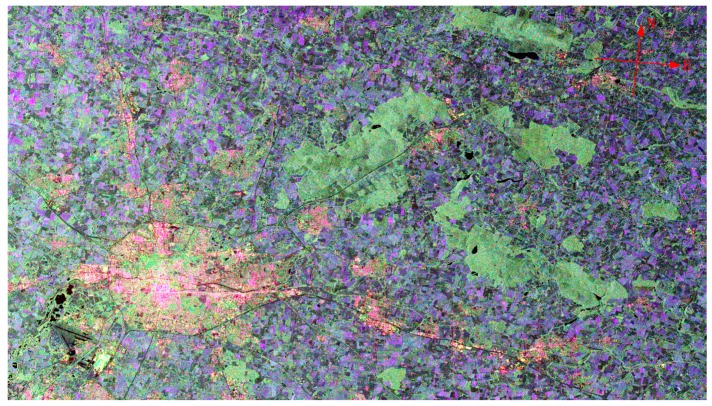
Q-mode image of Rennes city, France.

With its multi-polarization C-band SAR sensor, GF-3 can operate in 12 different observing modes, from high-resolution mode (1 m/10 km) to extremely-wide-swath mode (500 m/650 km), from single to quad polarization. Relying on the steering of the satellite platform, all observing modes can be realized with dual-side looking capability, which can reduce the revisit interval time. Figure 8 and Figure 9 give two example images of Rennes city, France, in P mode and Q mode respectively.

Applications of the GF-3 image products in the various fields [17,18,19,20] demonstrate its excellent performance and important value. Two follow-up satellites of GF-3 will be launched and put into operation before 2020, some design parameters will be improved to meet the feedbacks collected from end-users.

## Figures and Tables

**Table 1:** Observing modes of Gaofen-3 (GF-3).

Observing Mode	Incidence Angle (°)	Nominal Resolution ^1^ (m)	Nominal Swath (km)	Polarization
Spotlight (P)	20~50	1	10 × 10 ^2^	Single
Ultra-fine stripmap (UF)	20~50	3	30	Single
Fine stripmap (F)	19~50	5	50	Dual
Wide fine stripmap (WF)	19~50	10	100	Dual
Standard stripmap (S)	17~50	25	130	Dual
Narrow ScanSAR (NS)	17~50	50	300	Dual
Wide ScanSAR (WS)	17~50	100	500	Dual
Global observation (G)	17~53	500	650	Dual
Quad-pol stripmap (Q)	20~41	8	30	Quad
Wide quad-pol stripmap(WQ)	20~38	25	40	Quad
Wave (WV)	20~41	10	5 × 5 ^2^	Quad
Expanded incidence angle(E)	10~20	25	130	Dual
	50~60	25	80	Dual

^1^ After multi-look processing. ^2^ Image is discontinuous in azimuth, ×10 and ×5 refer to the azimuth length in one acquisition.

**Table 2:** Synthetic Aperture Radar (SAR) payload key parameters.

Parameter	Value
Center Frequency	5.4 GHz
Polarization	single, dual, quad
Antenna Size	15 m (A) × 1.232 m (E)
Number of Panels	4
Array Columns (Azimuth)	24
Array Rows (Elevation)	64 (32 in H, 32 in V)
Radio Frequency (RF) Peak Power	15,360 W
Average Power Consumption	8000 W max
Pulse Width	10–60 us (programmable)
Signal Bandwidth	2~240 MHz (programmable)
Transmit Duty Cycle	20% max
Receiver Noise Figure (at Module Input)	2.5 dB max
Receiver Channels	2
Pulse Repetition Frequency	1000~6000 Hz (programmable)
Sampling Frequency	533.33 MHz (IF sampling)
Quantization	8 bits
Data Compression	Block Adaptive Quantization (BAQ) 3 bits, BAQ 4 bits, High-4 bits, 8 bit (Selectable)
Output Data Rate	1280 Mbps max

**Table 3:** SAR performance.

Parameter	Properties	Comments
Polarization isolation	≥35 dB	
Polarization amplitude unbalance	≤0.26 dB,	Fixed bias compensated
Polarization phase unbalance	≤4.15°	Fixed bias compensated
Noise Equivalent Sigma Zero (NESZ)	≤−19.5 dB	P, UF, F, WF, Q, WV modes
	≤−21.3 dB	S, NS, WS, G, WQ, E modes
Azimuth ambiguity	≤−20.1 dB	P, UF, F, WF, Q, WV modes
	≤−18.8 dB	S, NS, WS, G, WQ, E modes
Range ambiguity	≤−20.0 dB	
Peak Side Lobe Ratio (PSLR)	≤−22.8 dB	P, UF, F, WF, Q, WV modes
	≤−20.5 dB	S, NS, WS, G, WQ, E modes
Integrated Side Lobe Ratio (ISLR)	≤−16.7 dB	P, UF, F, WF, Q, WV modes
	≤−15.3 dB	S, NS, WS, G, WQ, E modes
Radiometric resolution	≤3.50 dB	P, UF, F, WF, Q, WV modes
	≤1.89 dB	S, NS, WS, G, WQ, E modes
Absolute radiometric error	≤1.5 dB	
Relative radiometric error	≤1.0 dB	
